# Hospital and Institutional News

**Published:** 1920-05-01

**Authors:** 


### HOSPITAL AND INSTITUTIONAL NEWS.

### AN INVESTIGATION HOME.

The recently announced proposal to take over the mansion known as Grove House, Harrogate, for use as an investigation home for medical cases has, we understand, been practically decided upon. An outline of its aims and ideals was given by Sir Berkeley Moynihan, of Leeds, at the annual dinner of the Harrogate Medical Society, and apparently a private company which includes members of the Society is to be formed. Any scheme suggesting the correct use of team work and the realisation of the essential fact that the methods of clinical as well as experimental research are to be employed is worthy of commendation. In this case, it seems, a private company is going to do the pioneer work. We wish it success, but at the same time feel it is a pity that this type of organisation cannot emanate on the large scale from national authorities when the medical benefit is not for an isolated few, but for the nation in general.

### CONTROL OF THERAPEUTIC SUBSTANCES.

The Minister of Health has appointed a Committee to consider and advise upon the legislative and administrative measures to be taken for the effective control of the quality and authenticity of such therapeutic substances offered for sale to the public as cannot be tested adequately by direct chemical means. The Committee consists of the following members: Sir Mackenzie D. Chalmers, K.C.B., C.S.I. (Chairman), H. H. Dale, Esq., C.B.E., M.D., F.R.S. (head of Department of Biochemistry and Pharmacology under Medical Research Council), G. F. McCleary, Esq., M.D. (Medical Officer, Ministry of Health), A. B. Maclachlan, Esq. (Assistant Secretary, Ministry of Health), and C. J. Martin, Esq., C.M.G., D.Sc., F.R.C.P., F.R.S. (Director, Lister Institute of Preventive Medicine). The Secretary of the Committee is E. W. Adams, Esq., O.B.E., M.D., of the Ministry of Health, Whitehall, S.W. 1.

### THE CUP-TIE PROGRAMME.

The various professional football clubs subscribe more or less regularly to hospitals, but if we may judge from the extent of the gate-money taken at the matches they do not subscribe a great deal. There was a point of interest in connection with the Cup-tie football match at Stamford Bridge last Saturday in that the bulky programme, which was heavy with advertisements, contained one charitable appeal. At the top of every other page was the announcement that the Royal Hospital and Home for Incurables needs help. It would be interesting, if it were possible, to trace the effect of this reminder on the throng of spectators. Most of them ought to be subscribers to the Hospital Saturday Fund. But, alas! we fear that the Fund touches but a very small percentage of the works and workshops of London. In fact, one of the problems of the moment is to extend the scope of this Fund, not so much by a higher rate of contribution as by doubling and trebling the number of firms in which the Saturday collection is made. If the total sum collected by this Fund is compared to the number of "workers" in London it will be seen how few contribute to it. Is the football throng capable of responding to a general appeal? A modest attempt has been made to find out.

### THE PROGRESS OF THE LEEDS SCHEME.

The attempt to secure regular contributions from employers for the General Infirmary at Leeds has been successful. It is now an accomplished fact. So far, Mr. Braime's plan has been adopted by 100 firms, which have agreed to contribute at the rate of 2s. a year for each employee. Last week 4,000 more firms were approached by circular; and retail businesses are to be invited later. It is computed that there are at least 150,000 employers in Leeds and the district. If 2s. were subscribed for each of these by the employers £15,000 a year would be raised. The Workpeople's Hospital Fund is expected to raise, by its increased contributions, £20,000 a year. The yearly deficit of the General Infirmary is £25,000, so, in view of the progress already made, Mr. Charles Lupton's recent statement that the outlook is better does not seem exaggerated.

### DONCASTER ROYAL INFIRMARY SECRETARYSHIP.

Captain H. Kingsley Pearce, only surviving son of Mr. Edwin J. Pearce, Secretary-Superintendent of the Stockport Infirmary, and formerly Secretary of the Royal Hampshire County Hospital, has been appointed Secretary to the Doncaster Royal Infirmary. There were 150 applicants for this post, and four candidates were selected for an interview with the board. Captain Pearce was educated at Brighton College and Pembroke College, Oxford, and took his Commission in the Army shortly after the declaration of war, joining the London Regiment, with which he served in France. He transferred to the Royal Air Force in 1917, and when demobilised in November last was Adjutant of the Royal Air Force Station, Felixstowe.

### THE GLOUCESTERSHIRE MEDICAL SERVICE SCHEME.

The Ministry of Health has now formally approved the scheme, already mentioned in The Hospital, for the extension of the medical services in Gloucestershire. The extension of such services in county, that is to say, rural area, is a problem in itself. Dr. J. Middleton Martin, County Medical Officer of Health, favours a centre of treatment for each class of patient in every part of the country, even though the numbers of each class of case will necessarily be often small. The attendance of a consultant is part of the scheme, but it is not held that a specialist is necessary for routine examinations. The existing resources of the county consist of eleven cottage hospitals and 240 medical practitioners. The general hospitals, county council, and the doctors have agreed on the scheme, which is now approved. It proposes to open about fifty out-stations, somewhat resembling the present tuberculosis dispensaries, so that there shall be one within three miles of anywhere in the county. To these out-patients in all the classes of cases now provided with treatment out of public funds will come. The staff will consist of the local practitioners, and specialists from the hospitals will attend periodically and for cases reserved for them. Primarily the stations will deal with tuberculosis, venereal disease, school children, ex-Service men, and child-welfare. The county council will maintain the stations and pay the district nurse. Out of sums provided by the council, the hospitals will pay the medical officers. This county was early in the field, and the scheme has only been held back for want of official approval, which has been, as usual, a long time coming.

### HOSPITALS AND THE BUDGET INCREASE ON ALCOHOL.

The new spirit duties imposed by the Budget will increase the amount of duty to be paid on alcohol (rectified spirit) by an amount equivalent to 22s. 6d. per "proof" gallon. We have, on former occasions, drawn the attention of pharmacists and dispensers to the necessity of keeping records of the quantities of rectified spirit used in making medicinal preparations, so that the rebate allowed by the Government on this may be claimed. At the moment, it has not been stated that the rebate will be applicable to the new duty imposed, but it is confidently anticipated that the Chancellor of the Exchequer will follow the principle laid down, in former Budgets, of allowing a rebate on alcohol for use in *bona-fide* medical preparations. Hospitals may claim a rebate of about £4 13s. on every gallon of alcohol 90 per cent. used in the dispensary. We are informed that many of the smaller hospitals do not bother to claim the rebate, owing to the demand of the Excise authorities for careful records. Whilst the smaller hospitals buy tinctures and other alcoholic preparations from wholesale druggists (who themselves claim the rebate and fix a price accordingly), many preparations are made at these hospitals, involving the use of alcohol, and, if the rebate is not claimed, the expenses of the dispensing department are much in excess of what they need be. It should be mentioned that the procedure laid down by the Excise authorities is that duty must in all cases be paid on rectified spirit *before* delivery, the rebate having to be claimed later, on producing evidence to the authorities that this spirit has been used for medicinal preparations. Flavouring essences and tinctures are not regarded as "medicinal preparations," and, therefore, have to bear the full duties imposed.

### ALEXANDRA DAY.

Alexandra Day is to be celebrated on Wednesday, June 23, and an endeavour is to be made to obtain £50,000 in Greater London to help the hospitals and kindred charities. Sixty sub-committees have already been formed and are working in the Metropolis. A claim is especially made for the hospitals in recognition of the care they took of our sick and wounded men during the war when, although they "received a Government grant, it did not in many cases meet the expenses." The grant referred to, which by-the-bye was not accepted by all hospitals, was no doubt the payment made for maintenance, probably a better definition than that used by the Alexandra Day Committee in their announcement. We wish it could be brought more clearly to the notice of the public, who in the bulk think that their contributions are going into the coffers of the hospitals of London, so badly in need of support at the present time, that a large share of the collection does not go to hospitals at all, but to what are described, not very accurately, as kindred charities. Then, what does go to hospitals does not reach them all, as does, for instance, the Hospital Sunday Fund Collection, but only a chosen number. In the provinces there will be 500 centres this year, and altogether a record collection is expected.

### A MEDICAL SUPERINTENDENT'S WAR GRATUITY.

The Ministry of Health has informed the Edmonton Board of Guardians that Colonel Mort, medical superintendent of the hospital, is not eligible for a gratuity, and that the Army Council regrets that it cannot allow payment exceptionally. The Ministry also states that, as Miss Dowbiggin (the matron) is a "permanent Poor-law nurse," she is not entitled to the war gratuity for nurses, and that the Army Council is not prepared to agree that Army funds should be charged with such a gratuity.

### AUSTRALIA'S LEAGUE OF MERCY.

The annual distribution of the funds of the Hospitals' League of Mercy was made at the end of February. The amount distributed, writes our Australian correspondent, was £363. The League's funds are obtained by voluntary workers through the systematic canvass of the poorer classes for small donations, down to a penny a week. Though only founded in recent years, the total amount collected and distributed by the Hospitals' League of Mercy totals £6,378, this absolutely free from any deductions for expenses.

### WHAT TO DO WITH BAD CHILDREN.

When a parent is unable to control a child, an event for one reason or another not infrequent in these unsettled times, a visit for advice is invited to the Parent's Advisory Bureau of the Duty and Discipline Movement at 117 Victoria Street, S.W. This movement was started by the Earl of Meath in 1911, and has collected much useful information concerning the upbringing of children and the formation of character. It is thought that the scheme is likely to prove of value also to the medical profession, especially to those members who specialise in child psychology. After a parent has sought help a visit is generally paid to the home, or the child is brought to the Bureau. By dint of sympathetic questioning, the cause of the conduct complained of can generally be found. It occurs to us that persistent refractory conduct on the part of the child is generally caused by flabbiness of the parents, but no doubt the Bureau knows this as well as anyone and models its procedure accordingly.

### ISOLATION HOSPITAL PROVISION FOR CARMARTHENSHIRE.

THE Ministry of Health has intimated that it does not consider the scheme for an isolation hospital for Carmarthenshire, put forward at the recent inquiry by Dr. D. A. Hughes, the county medical officer of health, adequate to the needs of the area, and has asked for the adoption of a scheme for a hospital costing between £50,000 and £60,000—instead of the £15,000 allowed by Dr. Hughes—and the employment of a resident medical officer. A sub-committee of the Carmarthenshire Health Committee is, at the moment, going into the proposal for the purpose of reporting on it without delay to the County Council.

### THE JEWISH HOSPITAL.

THE work so far achieved by this institution was recorded at the recent annual court. The hospital known as the London Jewish Hospital was formally opened at Stepney Green on October 26 last. The average weekly number of out-patients is about 600, and it is hoped shortly to supply twenty-one beds in the two wards now being prepared. An *x*-ray department and a pathological laboratory have been provided. The chairman of the council, Dr. A. Goodman Levy, said the founders' intention was being observed, for most of the staff and patients were Jews, and could speak Yiddish, but where a Jew was not to be found sufficiently qualified, the appointment was given to some one else. The patients also, he added, are not wholly confined to Jews.

### A HOSPITAL'S APPEAL FOR "SERVANTS."

THE house committee of the Bradford Royal Infirmary has issued the following significant request:—"Owing to the impossibility of procuring domestic help . . . the nurses have been doing as much of the work pertaining to the unprocurable housemaids, wardmaids, and charwomen as their time and strength permit . . . an enormous effort which cannot possibly continue. An urgent appeal is now made for voluntary help (until such time as a sufficient domestic staff can again be procured) on the same lines as the Khaki Club, lately closed. The work may not be so attractive as that among the soldiers, but there is no doubt at all of its urgency." Names are thus entreated of women who can spare a few hours a week." Shifts of three hours will be arranged, beginning from 8 A.M., and "the duties will consist of sweeping, dusting, tidying, serving and clearing meals, and helping in the wards in the evening." The entreaty is signed by the president and honorary secretary of the ladies' committee. Perhaps no hospital appeal issued since the war is such a sign of the times as this. Will the young women who volunteered to do this work for soldiers volunteer for the sick? The answer has already been provided by the appearance of sixty volunteers for day work. Girl guides have offered to help in the evenings. This is timely, for the staff had been reduced, it is understood, to four.

### V.A.D.'S. AND OUT-PATIENTS.

SIR EDWARD STEWART, of the Executive Committee of the British Red Cross Society, discussed the future of the V.A.D. organisation at a meeting of the Chichester division of the Society at Bognor. Sir Edward said that the plan was for the joint bodies to act as reserves or auxiliaries to the Ministry of Health, as they had acted during the war to the Army Medical Service. V.A.D.s with nursing experience, if properly organised, might be useful to visit the homes of out-patients to help them to carry out the doctor's instructions. He thought that health visitors would welcome the help of trained V.A.D.s, who could also assist at crèches. He urged them, therefore, to stick to their detachments and to continue their training. It seems to us also that the hospitals should consider what work the V.A.D.s can do, for encouragement on both sides is needed if the co-operation is to be maintained.

### ORPHANS AND DOMESTIC SERVICE.

APPEALING for more subscribers to the Royal Female Orphan Asylum, Beddington, Surrey, Mr. Frederick Litchfield said that people believed girls were sent only to situations where three or four servants were employed. Some years ago that was the practice, because, he added, they were anxious to prevent a girl from becoming a drudge. But there was no rule; the inference seems to be that a suitable situation is welcomed whether in a small or large household. It would be interesting to know whether the impression, admittedly correct, has had anything to do with the complaint as to the shortage of subscribers.

### JOINT COUNCIL FOR SURGICAL INSTRUMENT TRADE.

THE first meeting of the new Joint Industrial Council for the surgical instrument and appliance industry was held last week at the Ministry of Labour. The Surgical Instrument Manufacturers' Association is represented by Mr. A. W. Down for the Employers, and the Amalgamated Instrument Society by Mr. H. Luckhurst for the men. These joint chairmen will preside alternately at the Council's meetings. For the present Mr. A. H. Bayes, of the Ministry of Labour, is acting as secretary.

### THE LIVERPOOL COUNCIL OF VOLUNTARY AID.

WITH apologies for its late appearance the January number of the Quarterly Paper issued by the Liverpool Council of Voluntary Aid, has now been published. It is an interesting index of the charitable organisations of the city, and contains a mass of what we may term committee news, together with a brief digest of recent Government Acts and publications. It draws attention to the large balances still possessed by war charities whose work is done, and discusses the legal question how properly to spend them. The cyprès solution has obvious difficulties, and may turn upon a definition which, when challenged in the courts, will be upset. The council therefore suggests, with the approval of the Charity Commissioners, that at the foot of all local appeals for temporary objects in the future, a note shall run to notify the public that any surplus balance will be allotted to the Liverpool Council of Voluntary Aid Fund for the benefit of Liverpool charities. Why does not the council, having done something to safeguard the future, issue a report of the present war charities which have an unexpended balance, state its amount, and thus set the existing state of affairs before the public. The Quarterly Paper would be the proper place for this, since it is intended to be read only by those directly interested.

### FREE BEDDING FOR CONSUMPTIVES.

The Greenwich Borough Council's Health Committee, holding it to be very undesirable that persons suffering from tuberculosis should sleep with the healthy, recommends, under the Council's arrangements for the treatment of tuberculosis, the provision for each necessitous case of a single bed, mattress and flock overlay. Is flock to have official benediction? Always avoidable, beyond institutional supervision it should be banned.

### THE AFFILIATION OF DISPENSARIES.

For some time past the Hammersmith Borough Council has been trying to affiliate its tuberculosis dispensary with a hospital, and the authorities of the West London Hospital are ready to arrange with the Council to refer cases to the hospital for consultation if the Council will pay one guinea per case. The Tuberculosis Officer expects to refer fifteen or twenty cases a year to the hospital. The Council favours the plan.

### PRESENTATION TO A HOSPITAL MATRON.

Miss Timbrell, who lately resigned the post of matron at Lowestoft and North Suffolk Hospital, in order to become matron of "a new hospital in Kingsway," London, has been presented with an illuminated album containing the names of 467 subscribers and a cheque for £163. The inscription records Miss Timbrell's connection of ten years with the hospital. The nursing staff also made its own present to her. Miss Timbrell was in the South African War, went to the Gold Coast, and from the Gold Coast to Siam. After three years in Siam she returned to England and became a senior sister at Carlisle Hospital, from which she went to Lowestoft in 1910.

### GOOD WORK AT MAESTEG.

The Committee of the Maesteg and District General Hospital, Maesteg, Glam., have adopted plans for extensive additions and improvements to the existing building, at an estimated cost of £30,000 for building alone. The extensions include a women's ward, children's ward, seven small wards of one or two beds each, a new operating theatre (the existing one to be used for septic cases), an $x$-ray and massage department, and additional quarters for nursing and domestic staff. The improvements include electric lighting and heating. The hospital was given up entirely during the war to the use of the Red Cross Society for the treatment of wounded soldiers, and it still treats ex-soldiers. A Women's Guild has just been established in connection with the hospital, and the members have laid down an ambitious programme. They hope to be in a position to assist largely in the furnishing of the new quarters when finished, and are at present maintaining the hospital in linen, etc.

### A JOINT CONTRIBUTORY FUND FOR HARROGATE.

Mr. George Ballantyne, the Secretary of the Harrogate Infirmary, has drafted a contributory scheme which is now being considered by the firms of the neighbourhood. To be known as the Harrogate and District Employers and Workers' Joint Contributory Fund, the plan is for each employee to subscribe 2d. a week (1d. in the case of a woman), and for the employers to give 7d. and 5d. respectively per quarter. If accepted, as we trust it will be, the scheme will begin in July next. In the meantime each employer is to lay the matter before his workpeople. We shall be glad to learn what success the proposal meets with, and the income which will result from the extent to which it is adopted.

### RETIREMENT OF A HOSPITAL SECRETARY.

The recent retirement of Mr. J. A. Dees from the secretaryship of the Northern Counties Hospital for Consumption occurs at a time when the accounts show a balance in hand. This is a welcome and unusual state of affairs at present, of which Mr. Dees may be proud. He has held his post for eighteen years, and has now been appointed a member of the committee.

### THIS WEEK'S DRUG MARKET.

The break in prices which recently set in is more noticeable, and buyers are holding off in the hope that we are at the beginning of a general downward movement. In some cases prices had, no doubt, advanced to an unjustifiable figure, and in such cases as these the downward movement is especially noticeable now that the demand has to some extent been met and there is less money available for speculative purposes. Price movements, whether upwards or downwards, are apt to be contagious, and it is therefore not improbable that the break in values will become more comprehensive. The increase in the spirit duties will not affect the prices of spirituous medicinal preparations, since the usual rebate is to be allowed; but there is a heavy advance in the prices of spirituous preparations which are not classed as medicinal, as, for instance, tincture of orange. In addition to menthol, camphor, and Japanese peppermint oil, the following are among the articles which have a downward price tendency:—star aniseed oil, cloves, clove oil, castor oil, cod-liver oil, senega root, podophyllum root, lemon oil, cream of tartar, cascara sagrada, and hexamine. The best advice that can be given at the moment is to use special caution in buying.

